# Physiological performance of *Kazachstania unispora* in sourdough environments

**DOI:** 10.1007/s11274-021-03027-0

**Published:** 2021-04-21

**Authors:** Dea Korcari, Giovanni Ricci, Claudia Capusoni, Maria Grazia Fortina

**Affiliations:** 1 Dipartimento di Scienze per gli Alimenti, la Nutrizione e l’Ambiente, Università degli Studi di Milano, Milan, Italy; 2 Department of Food, Environmental and Nutritional Sciences, University of Milan, Via Celoria 2, 20133 Milan, Italy

**Keywords:** Non-conventional yeasts, *Kazachstania unispora*, Sourdough fermentation, Stress tolerance

## Abstract

In this work we explored the potential of several strains of *Kazachstania unispora* to be used as non-conventional yeasts in sourdough fermentation. Properties such as carbohydrate source utilization, tolerance to different environmental factors and the performance in fermentation were evaluated. The *K. unispora* strains are characterized by rather restricted substrate utilization: only glucose and fructose supported the growth of the strains. However, the growth in presence of fructose was higher compared to a *Saccharomyces cerevisiae* commercial strain. Moreover, the inability to ferment maltose can be considered a positive characteristic in sourdoughs, where the yeasts can form a nutritional mutualism with maltose-positive Lactic Acid Bacteria. Tolerance assays showed that *K. unispora* strains are adapted to a sourdough environment: they were able to grow in conditions of high osmolarity, high acidity and in presence of organic acids, ethanol and salt. Finally, the performance in fermentation was comparable with the *S. cerevisiae* commercial strain. Moreover, the growth was more efficient, which is an advantage in obtaining the biomass in an industrial scale. Our data show that *K. unispora* strains have positive properties that should be explored further in bakery sector.

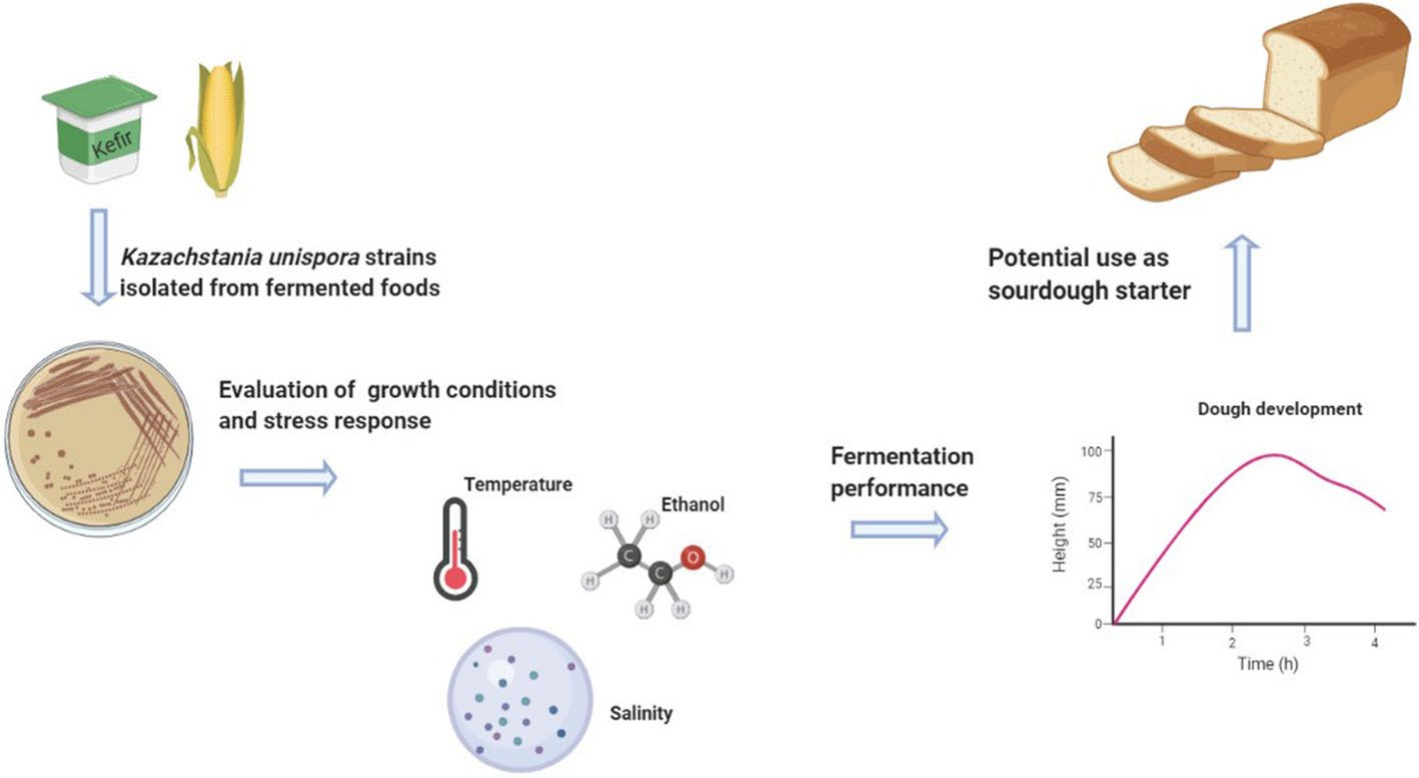

## Introduction

In the last decade, a great attention has been paid to design new microbial starters for food sector. Particularly, the demand for products with peculiar aroma profiles and improved nutritional properties led to a renewed interest into the characterization of non-conventional microbial cultures selected from spontaneous food fermentations (Steensels and Verstrepen 2014; Aslankoohi et al. 2016). The use of the sourdough process is one of the oldest spontaneous biotechnological processes in cereal food production. In sourdough preparations the autochthonous microbiota, composed of yeasts and Lactic Acid Bacteria (LAB) confers positive features to the final product, positively influencing the technological, nutritional and organoleptic properties and implementing the shelf-life of the bread (De Vuyst et al. 2016; Martorana et al. 2018). For these reasons there is a growing interest in sourdough preparations and in investigating the potential of the autochthonous microflora found in spontaneous sourdough fermentations. However, while the study and the use of LAB species has received considerable attention, the study and the use of non-conventional yeasts for bread dough fermentation has received relatively little attention. Although these yeasts do not always have the leavening ability of bread yeast, they can strongly contribute to improve the nutritional characteristics and the flavor profile of the product. They also show a higher tolerance towards stressful conditions such as pH, osmotic and oxidative stress (De Vuyst et al. 2016; Zhou et al. 2017).

*Kazachstania unispora* (formerly *Saccharomyces unisporus*) (Bhattacharya et al. 2013) is a non-conventional species of yeast, belonging to a genus “*Saccharomyces *sensu lato species”, which contains more of 40 different species, isolated from several habitats (Carbonetto et al. 2018). *K. unispora* has been found in traditional dairy products, and represents a characteristic species of the autochthonous microbial population of kefir, where it seems to have an active role as probiotic (Marsh et al. 2013; Bourrie et al. 2016). The species has also been found in sourdoughs, albeit to a lesser extent. Two groups of sourdough *Kazachstania* species have been defined (Carbonetto et al. 2018). The representative species of the first group is *K. exigua*, the most frequently cited sourdough species in the literature. Within the second group, *K. unispora* remains poorly characterized.

*K. unispora* is able to ferment galactose but not lactose; its frequent presence in dairy products could be due to this ability, thus not competing with lactose fermenting bacteria (Montanari et al. 1996). For the same reason, *K. unispora* could be adequate to be used as selected culture in sourdough fermentation, because of its inability to ferment maltose. Indeed, maltose negative yeasts could establish a more stable consortium with LAB in sourdoughs because of a lack of competitiveness for the carbon source (De Vuyst and Neysens 2005; Venturi et al. 2012).

Besides the fermentative role, *K. unispora* produces a number of metabolites with an important impact on the sensory profile of the product, but their role in the nutritional properties are still to be studied (Bhattacharya et al. 2013). Some studies indicate the ability of strains of *K. unispora* to accumulate high amount of palmitoleic acid (Nabi et al. 2016; Gientka et al. 2017). This characteristic may be of particular interest, since the supplementation with this mono-unsaturated fatty acid has been linked to a diminished risk to develop cardiovascular diseases (Griel et al. 2008).

*Kazachstania unispora* do not seem to pose human health risks: strains of this species isolated from kefir have been associated to low virulence profiles (Lim et al. 2019). The observed resistance to the antifungal fluconazole may be regarded as intrinsic to the species, not easily transmissible, as reported for *S. cerevisiae* strains, showing reduced susceptibility to most azole agents (Kontoyiannis and Rupp 2000; Lim et al. 2019). Furthermore, to the best of our knowledge, there are no reports of infections caused by the yeast *K. unispora*, and the species is included in the Inventory of microbial food cultures with safety demonstration in fermented food products (Bulletin of the International Dairy Federation 2018).

This study aimed to be a further exploration of the potential of the species *K. unispora*, through the evaluation of properties not yet deepened. Specifically, we characterized several *K. unispora* strains with the aim to use selected strains as alternative baking yeasts.

## Materials and methods

### Strains and growth conditions

*Kazachstania unispora* strains were previously isolated from fermented maize bran (11 strains named KM 1–11) (Decimo et al. 2017) and from artisanal kefir grains (5 strains named KK12-16). A commercial *Saccharomyces cerevisiae* strain (named SC) was purchased from AL.NA Srl (Turin, Italy) and used for comparison.

Strains were routinely subcultured in YPD broth at pH 6.0 and grown at 28 °C, either in static or shaking conditions. The composition of the medium is as follows (g L^−1^): yeast extract 10, peptone 20, glucose 20. Pure cultures were maintained on agar YPD at 4 °C for short term storage, and in YPD broth supplemented with glycerol (15% v/v) at − 80 °C for long term storage. The cultures are deposited in the Microbial Collection of the Department of Food, Environmental and Nutritional Sciences, University of Milan, Italy.

### Growth on different carbohydrate sources

Cell growth on YP medium supplemented with 1% (w/v) of glucose, maltose, fructose and sucrose was monitored by optical density at 600 nm (OD_600_) using a plate reader (Biotek, Vermont, USA). The plate reader was run in discontinuous mode, with absorbance readings performed in 30 min intervals and preceded by 30 s shaking at medium speed. Cultures were grown in independent triplicates and the resulting growth data were expressed as the mean of these replicates. Carbohydrates were dissolved in water, sterilized by filtration (0.2 µm filter size) and then added to autoclaved YP. Cells from pre-cultures grown in YPD broth were used as inoculum: they were harvested during the exponential phase of growth by centrifugation, washed twice with a saline solution (NaCl 0.9% w/v) and inoculated at 1% (v/v) (starting OD_600_ between 0.06–0.08).

### Tolerance to different environmental factors

Pre-cultures of the strains, obtained as reported above, were used as inoculum (10^5^ CFU/ml) to test the ability of the strains to tolerate different types of stressors. The evaluation of the growth was done by OD_600_ determination after 24–48 h of incubation at 28 °C in static conditions, in comparison with the growth in standard conditions. All tests were conducted in triplicates.

#### Temperature and pH tolerance

All strains were investigated for their ability to grow in YPD broth at different temperatures (25°, 30°, 37° and 42 °C) and at different pH of the medium (2.5, 3.0, 3.5 and 4.0).

#### Osmotolerance

Yeast strains were cultured in YPD broth containing 10 and 30% glucose or fructose and incubated for 48 h. We also evaluated the tolerance toward two types of stressors: a low pH (pH 3.0) and low/high osmotic stress (glucose; 10% and 30%). Further, the growth of strains after incubation in YPD broth added with 2 and 6% NaCl was investigated.

#### Organic acids and ethanol tolerance

The ability of the strains to grow in high organic acid concentrations was tested in YPD broth at pH 5, supplemented with 1% (v/v) lactic acid, 1% (v/v) acetic acid or a mixture of both organic acids (0.5% + 0.5% v/v).

The resistance to ethanol was assessed by adding to the medium 4, 6 or 12% of ethanol (v/v).

### Glucose and ethanol assays

A representative strain (KM 11) was chosen for testing glucose and ethanol concentrations in the supernatants of the cultures grown both in static or shaking conditions. The assays were carried out in triplicate using commercial enzymatic kits (catalog no.1 076251 035, 1 0176290 035; Hoffmann La Roche, Basel, Switzerland). Each batch was inoculated with 10^5^ UFC/mL of overnight grown, twice washed cells.

### Rheofermentometer assay

The performance in fermentation of dough of the representative strain KM11 was measured in a rheofermentometer assay, using a Chopin F4 Rheofermentometer (Chopin Technologies, Villeneuve-la-Garenne Cedex, France) at 30 °C for 8 h. The height reached by the dough was recorded for the *K. unispora* strain and for the commercial *S. cerevisiae* strain as a comparison.

### Statistical analysis

Statistical analysis was performed using GraphPad Prism 8 (v. 8.4.3, GraphPad Software Inc., California, USA). Results are expressed as mean ± SD and are analyzed with two-tailed unpaired T-test. The significance level is indicated with n.s. for non-significant differences, one asterisk (*) for p < 0.05, two (**) for p < 0.01 and three (***) for p < 0.001. Post-hoc Tukey’s HSD test was performed with p < 0.05 and different letters indicating significant differences.

## Results

### Growth and carbohydrate sources utilization

**Fig. 1:**
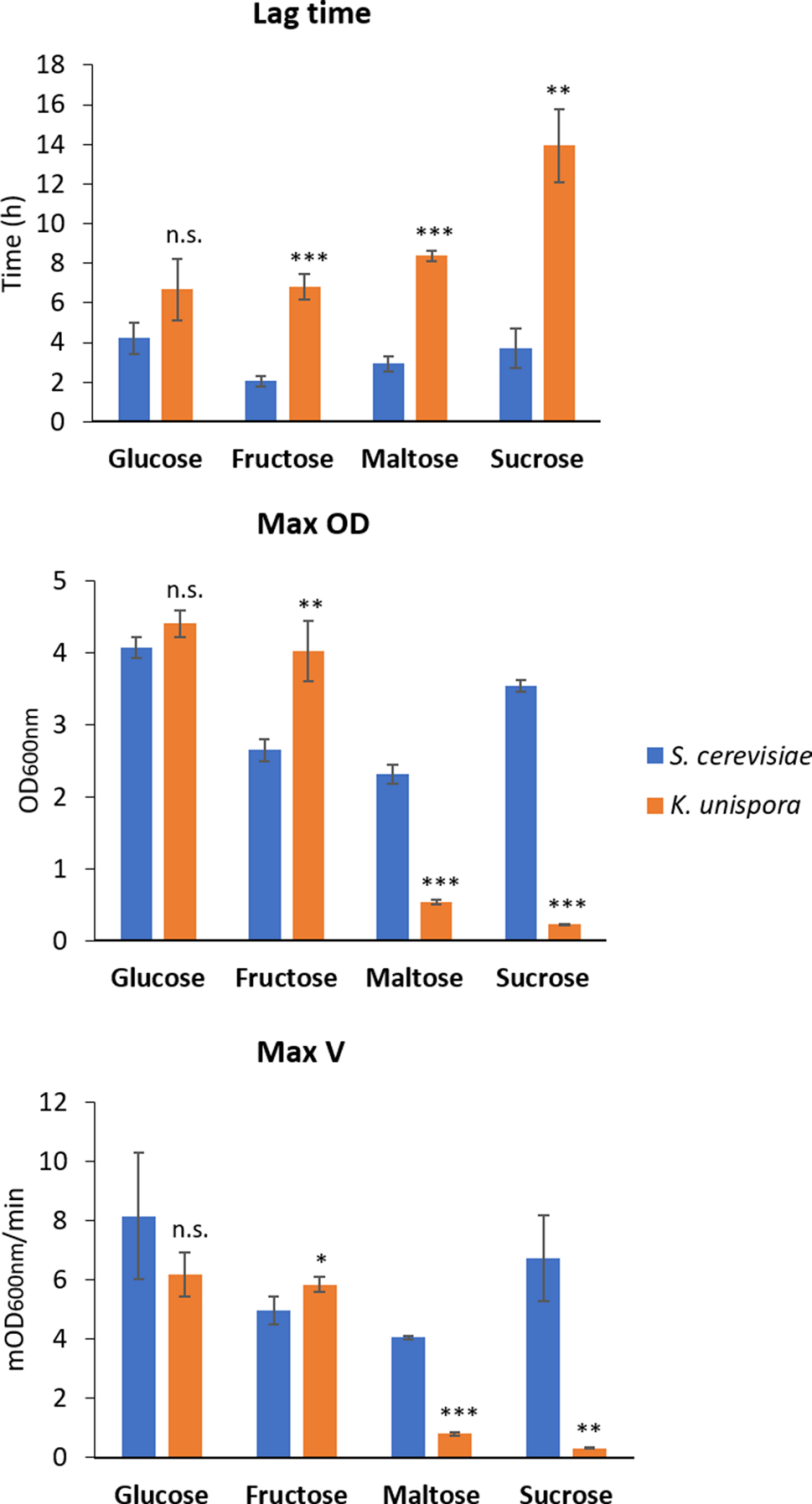
Lag time (h), Max OD and MaxV (mOD/min) of *S. cerevisiae* SC and *K. unispora* KM11 with different carbohydrate sources, incubated at 30 °C. Results are expressed as mean of three independent experiments ± SD. Asterisks indicate the significance level (* for p < 0.05; ** for p < 0.01; *** for p < 0.001; n.s. for non-significant p-value)

To characterize growth and carbohydrate sources utilization patterns of *K. unispora* strains*,* the yeasts were cultivated in YP medium supplemented with sugars that are present in flour. The *K. unispora* strains are characterized by rather restricted substrate utilization (Fig. 1): only glucose and fructose supported the growth of the strains. Sucrose is not utilized by the strains, indicating a lack of invertase activity. However, although the lag time is higher, compared with the *S. cerevisiae* strain, the final OD reached in presence of fructose is also higher (cell density 31% to 115% higher).

### Tolerance to different environmental factors

**Fig. 2:**
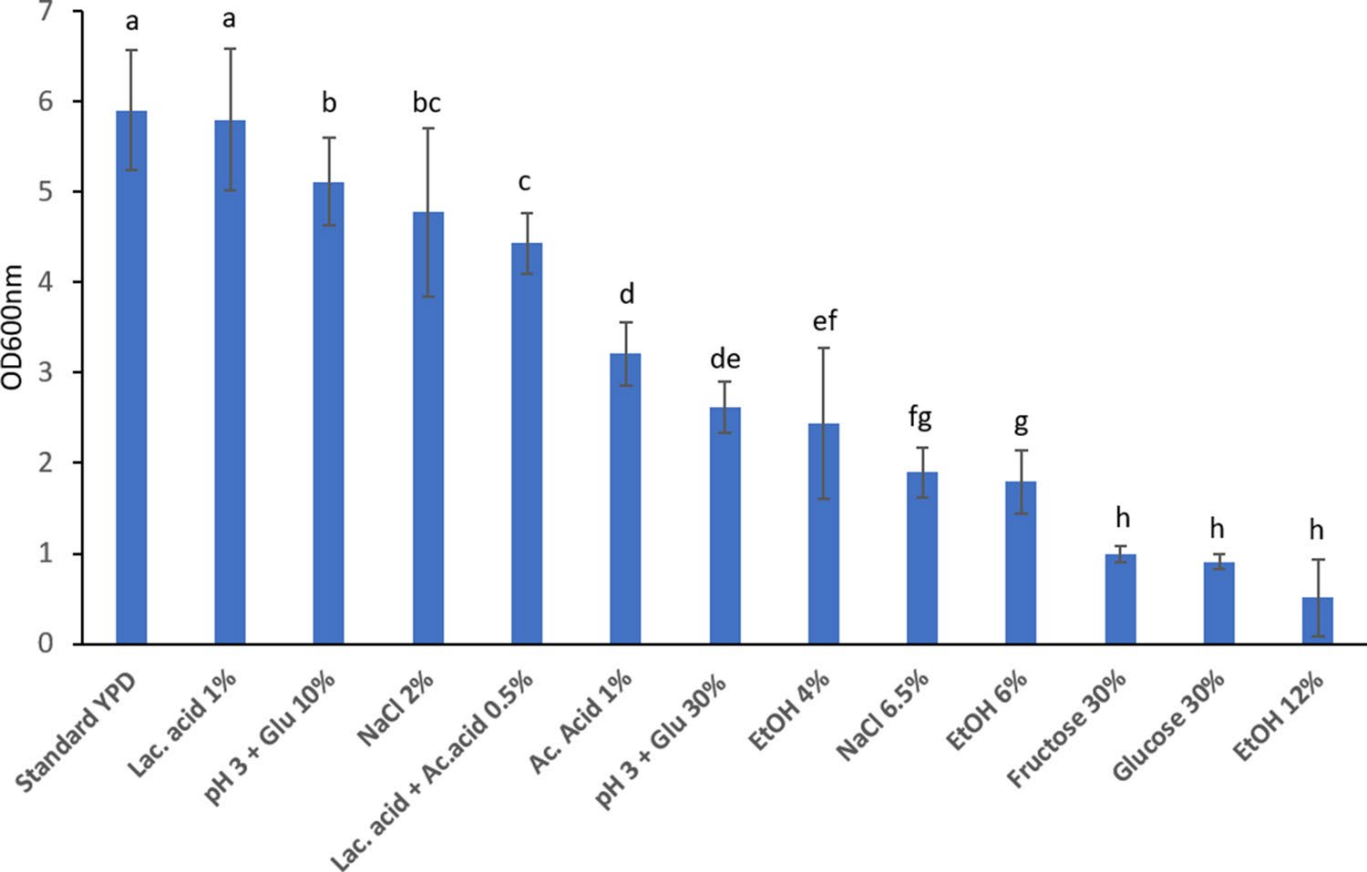
Stress response of *K. unispora* strains grown in different stress conditions, compared to the growth in standard YPD medium at 30 °C. Results are expressed as mean of three independent experiments ± SD, and different letters indicate significant differences (post-hoc Tukey’s HSD test, p < 0.05)

The growth of the *K. unispora* strains was checked under different stress condition. As reported Fig. 2 the strains could withstand the series of stress and were able of adapting to the conditions tested.

All strains grew well in the pH range 3.0–6.0. In response to temperature change, all the strains were able to grow at temperatures ranging from 25 °C to 30 °C. At 37 °C the residual growth was very low, between 4 and 12% for all strains. No growth was recorded at 45 °C after 48 h, furthermore no strain was able to start again the growth when transferred at 28 °C. The ability to grow at low pH and temperature values is a desirable property for their potential use in type I sourdoughs, characterized by low incubation temperatures and continuous back slopping with a pH value of 3–4 reached by the growth of LAB.

The presence of ethanol had a great impact on the growth of the strains. Only moderate growth was observed in presence of 4% and 6% of ethanol; however, some level of growth was observed for 44% of the tested strains in presence of 12% of ethanol.

Regarding the tolerance to organic acids, the performance varied when lactic or acetic acid was tested: in presence of 1% of acetic acid, the growth was about 50% of the standard conditions whereas lactic acid did not influence the growth of the strains. The two organic acids did not appear to have any synergistic effect in inhibiting the strains’ growth, as they reached intermediate values of growth when compared to the single acids.

The strains were also screened for the ability to adapt to osmotic stress, by evaluating the growth in presence of high sugar and salt concentration. In presence of 2% NaCl, a condition similar to concentrations used in bread-making, all strains grew well, with most of them reaching values of 60% of residual growth. A higher concentration (6.5%) reduced the ability of all strains, while still maintaining noteworthy growth levels.In presence of 30% of glucose or fructose, the growth ranged between 11 and 22% of the standard conditions. At a pH value of 3, the growth in presence of 30% of glucose was more efficient, and was 1.8 to 3.7 times higher than the growth at pH 6.0 and 30% glucose.

### Glucose and ethanol assays

**Fig. 3:**
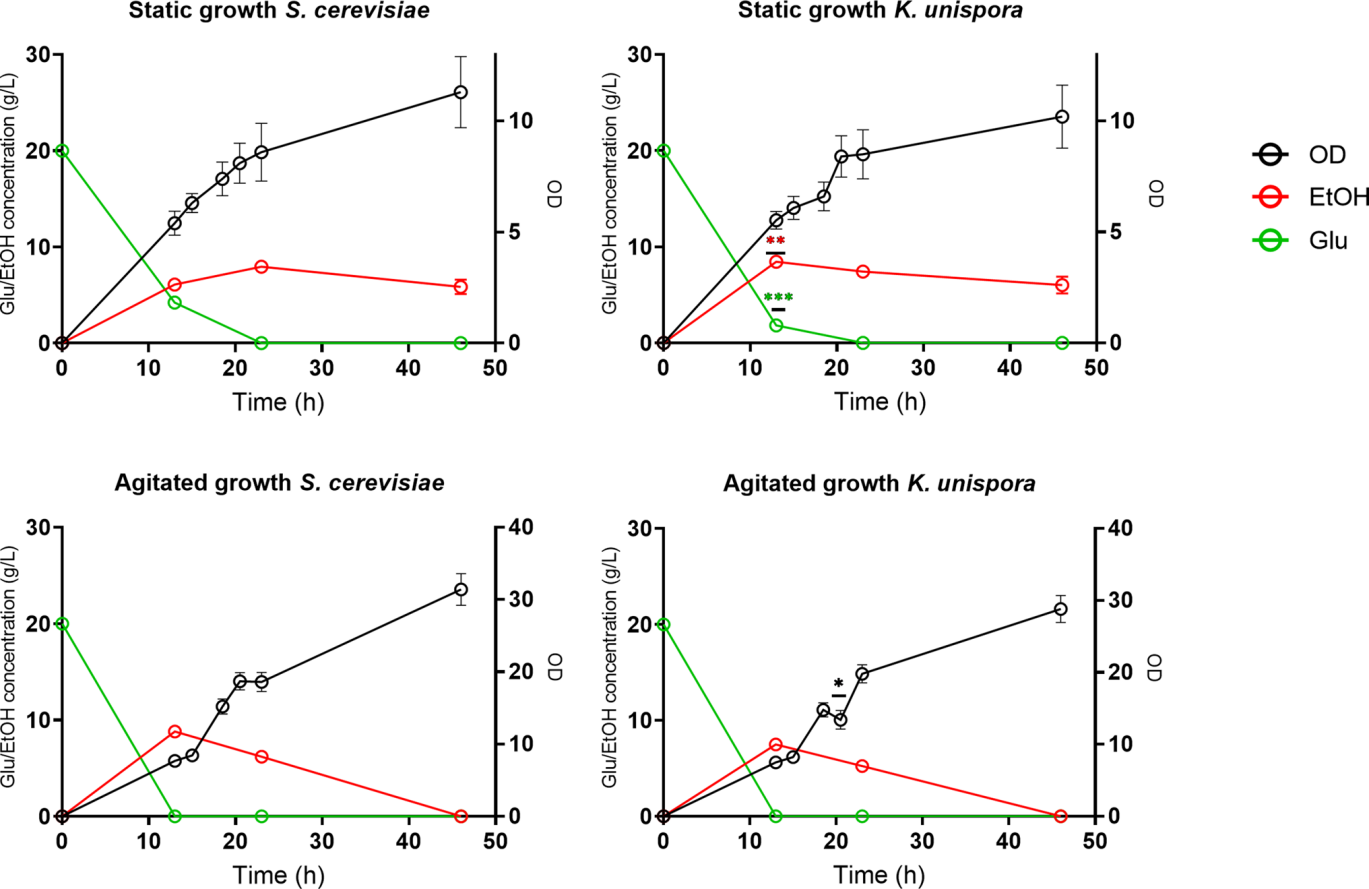
Glucose consumption, ethanol production and OD of *S. cerevisiae* SC and *K. unispora* KM11 grown in static or agitated conditions. Results are expressed as mean of three independent experiments ± SD. OD (Black); EtOH (Red); Glucose (Green)

One strain of *K. unispora* (KM 11) was used to compare the performance in fermentation with the *S. cerevisiae* strain. The results are reported in Fig. 3. The behavior of the two strains is similar: the glucose is rapidly consumed and is not detectable after 13 h when the strains were grown in agitating condition, and the ethanol concentration was at a peak level of 8.8 g/L for *S. cerevisiae* and 7.9 g/L for *K. unispora*. In the subsequent hours ethanol was consumed and cell growth continued, until it became undetectable. In static conditions the dynamics were slower; the peak of ethanol production was reached after 23 h of fermentation for *S. cerevisiae*, whereas it overlapped with the agitating growth for *K. unispora*. The maximum concentration of ethanol also was comparable between the two conditions, but it was poorly consumed after 46 h. The final cell density recorded was comparable for both strains (1.82 × 10^7^ CFU/ml for *K. unispora*, 1.79 × 10^7^ CFU/ml for *S. cerevisiae*) and 2–3 times lower than that recorded in agitating conditions (4.44 × 10^7^ for *K. unispora*, 4.78 × 10^7^ CFU/ml for *S. cerevisiae*).

### Rheofermentometer assay

**Fig. 4:**
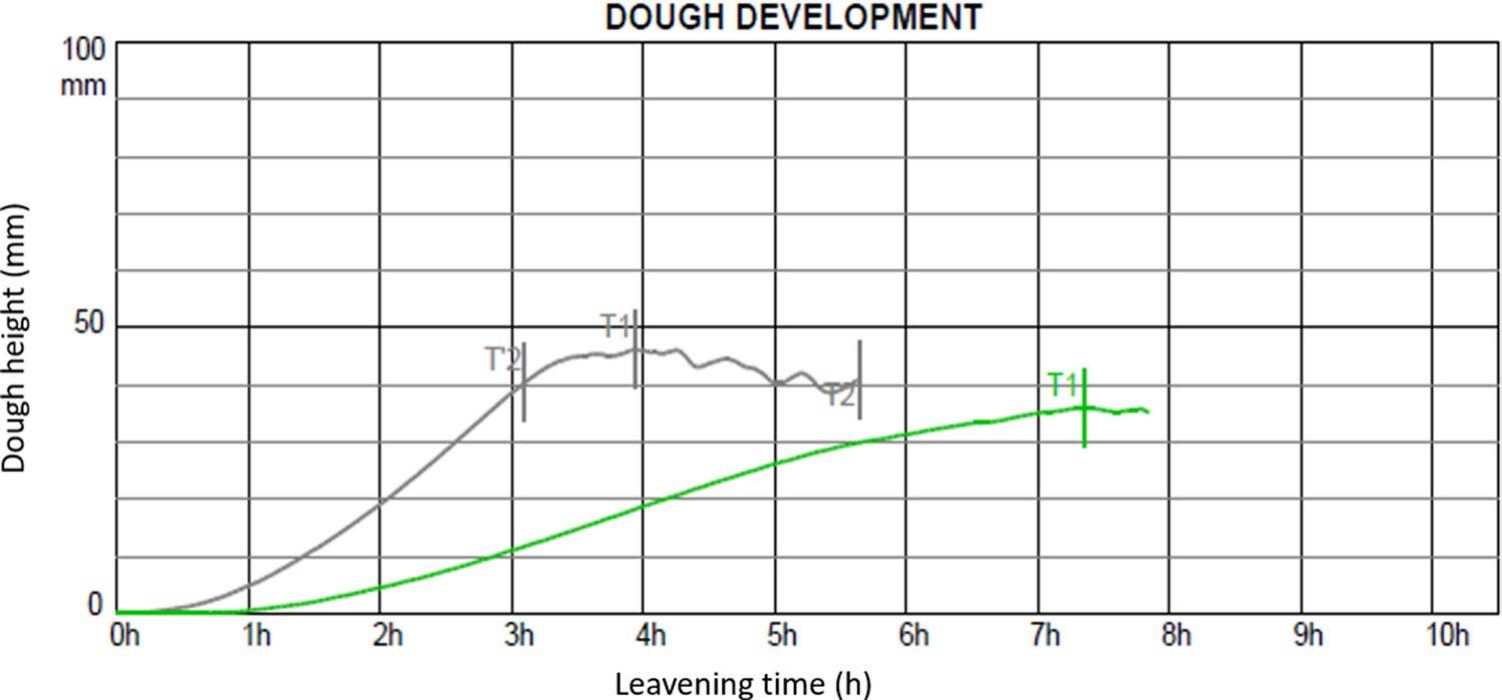
Rheofermentometer analysis showing the development of the dough (mm) leavened with *S. cerevisiae* SC (Black) and *K. unispora* KM11 (Green)

As expected, due to the inability of the *K. unispora* strain to ferment maltose, the leavening of the dough is slower compared to the commercial *S. cerevisiae* strain, as can be seen in Fig. 4. Whereas *S. cerevisiae* reaches the maximum height after 4 h, the dough leavened with *K. unispora* does not appear to reach the maximum height at the end of the 8 h. The height reached by *K. unispora* is 35.9 mm that is lower compared to the 46.2 mm reached by *S. cerevisiae*.

## Discussion

In this study we tested some properties not yet known of *K. unispora* strains, with the aim to evaluate their potential role as non-conventional yeast species in sourdough fermentation. Nowadays, there is an increasing interest of revisiting the starter cultures, including the autochthonous microbial population, in an attempt to improve the peculiar characteristics, the quality and the safety of the final products. In this context, it is important to explore and to study the potential of new strains from diverse ecological niches for industrial relevant uses.

In relation to the baking sector, together with the industrial bread production obtained using the commercial baker’s yeast *Saccharomyces cerevisiae,* commercially sourdoughs containing selected yeasts and LAB strains are also available. If the LAB proposed as sourdough starters are well defined, the search for non-conventional yeast species is still in progress. In this context, we deepened the knowledge of several strains of *K. unispora.*

Our results show that *K. unispora* are adapted to a sourdough environment, since the strains tested are able to grow in conditions associated with it, such as high osmolarity, high acidity and the presence of organic acids, ethanol and salt. The growth in lactic acid was more efficient when compared to acetic acid, which is in accordance with literature reports showing that lactic acid has a higher MIC compared to acetic acid. Because of the higher pK_a_ value of acetic acid, at any acidic pH value there is more undissociated acetic acid than lactic acid, that penetrates the membrane and dissociates inside the cell, lowering the intracellular pH (Narendranath et al. 2001).

**Fig. 5:**
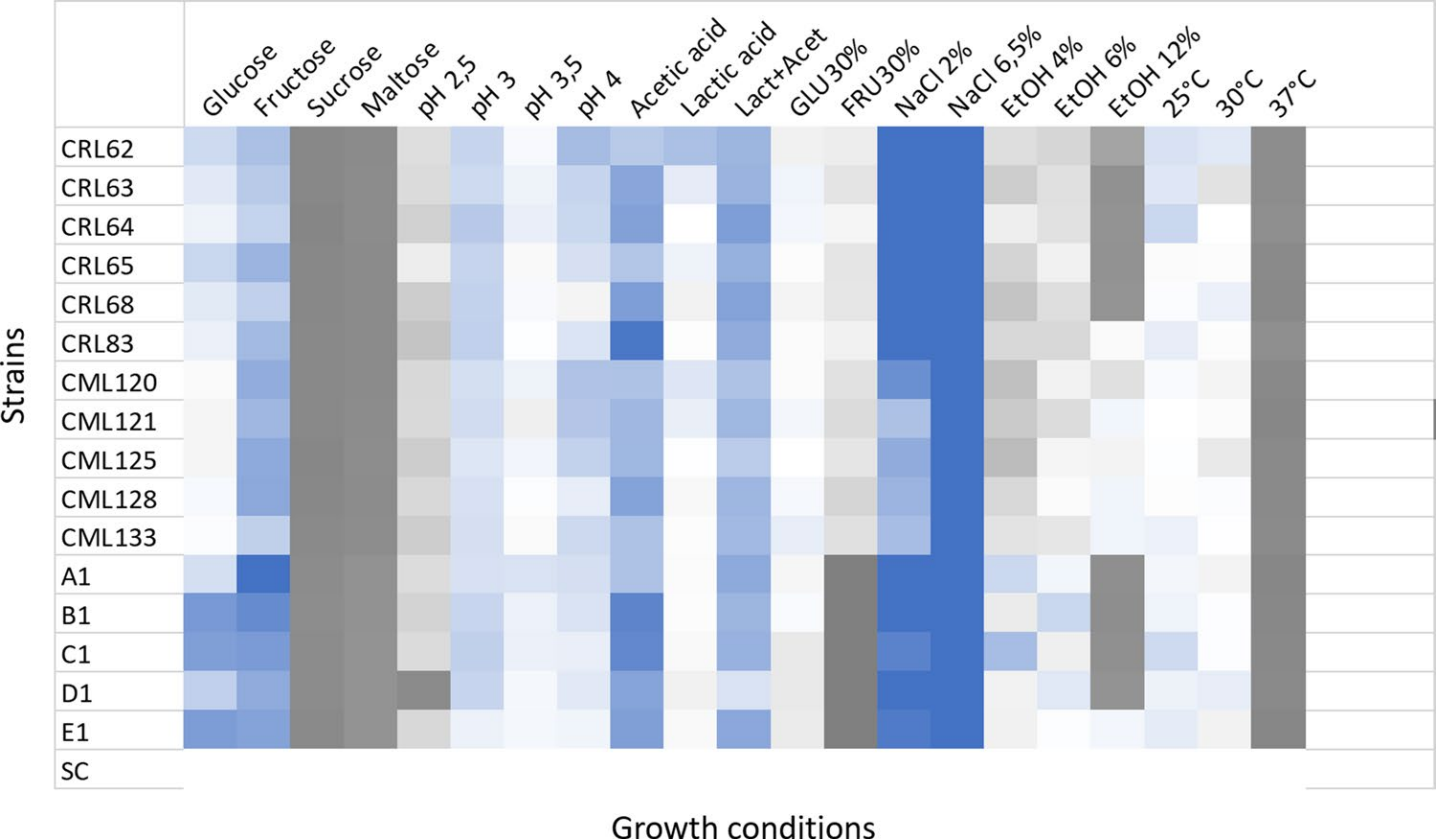
Heatmap of the performance of *K. unispora* strains relative to *S. cerevisiae* in stress conditions. Scale shows the proportion of growth of *K. unispora* compared to *S. cerevisiae*: 0 indicates no growth, 1 indicates equal growth,  > 2 indicates the growth is at least twice as much

Moreover, when the performance of the *K. unispora* strains was compared to the commercial strain of *S. cerevisiae* (Fig. 5) it was possible to note that strains of *K. unispora* performed better under some stress conditions, in particular in high salinity or in presence of acetic acid, with all strains outgrowing *S. cerevisiae*; the commercial strain grew very poorly in the medium added with 6% NaCl and showed a moderate growth in presence of acetic acid. The tolerance to low pH values is similar, whereas at pH 4 (the pH value typical of type I sourdoughs) the growth is more efficient for *K. unispora* strains. In combined pH and osmolarity conditions, almost all *K. unispora* strains outgrow the *S. cerevisiae* strain*.* On the contrary, *S. cerevisiae* is able to resist to high ethanol concentrations, outgrowing most *K. unispora* strains, although some strains achieved a comparable growth even at 12% of ethanol. The lower growth rate in presence of high sugar concentration is a well-known phenotype of *S. cerevisiae* and other Crabtree positive yeasts, that, when exposed to high sugar concentrations, shift their metabolism to aerobic fermentation: the fact that the sugar is mainly used to produce ethanol and its accumulation, together with other metabolites such as weak acids, limits the growth of the yeast. (Dashko et al. 2014). A similar mechanism could be hypothesized for *K. unispora*.

The *K. unispora* strains are maltose-negative, but this characteristic might be not negative in sourdoughs: their inability to ferment maltose makes them suitable strains in stable consortia with maltose consuming LAB, which supply the fermentable sugars used by the yeast in fermentation (Gobbetti 1998; De Vuyst et al. 2009; Carbonetto et al. 2020). *K. unispora* strains are also unable to ferment sucrose. This characteristic is not advantageous when molasses are used for the industrial production of baking yeast starter cultures. The cost and efficient use of carbon sources are crucial for economical production of yeast biomass. However, alternative low cost substrates could be used for *K. unispora*, such as glucose syrups derived from starch hydrolysis, high fructose corn syrups (HFCS) or by-product of corn-starch extraction process (Spigno et al. 2009; Yu et al. 2015). The performance in fermentation was inferior compared to *S. cerevisiae*, but the growth was more efficient, which is an advantage in obtaining biomasses in an industrial scale. Moreover, although *K. unispora* is outperformed in a straight dough context, its longer fermenting time and the ability to act in synergy with LAB can be used in sourdough production, where, especially for a type I sourdough, longer leavening times are typical. Preliminary results obtained in our laboratory, by testing in cocultures *K. unispora* and different LAB strains usually found in sourdoughs, indicate no significant differences in yeast/LAB population density. For these reasons, it is possible to hypothesize the creation of mixed cultures consisting of *K. unispora* and selected LAB strains for sourdough bread making. Further experiments are in progress in our laboratory, testing this microbial association and evaluating the characteristics they provide to the final bread product.
